# Cryo-EM Structures of Apo, Agonist- and Antagonist-Bound Heteromeric Kainate Receptors

**DOI:** 10.64898/2025.12.02.691905

**Published:** 2026-06-27

**Authors:** Changping Zhou, Guadalupe Segura-Covarrubias, Ashlee M’Kay Hoffman, Nami Tajima

## Abstract

Kainate receptors (KARs) mediate excitatory synaptic transmission and regulate neurotransmitter release. In the central nervous system, KARs predominantly exist as heterotetramers comprising low-affinity (GluK1–3) and high-affinity (GluK4–5) subunits, with GluK2/GluK5 being the most abundant. To elucidate their conformational transitions, we determined the cryo-electron microscopy (cryo-EM) structures of GluK2/GluK5 KARs in the apo and distinct ligand-bound states. The apo structure revealed compact packing with extensive inter-subunit interactions between the ligand-binding domains (LBDs) beyond the conserved D1-D1 upper-lobe contacts. The glutamate-bound structure exhibited enhanced packing that stabilized the desensitized conformation through increased intersubunit contacts relative to the homomeric KARs, indicating that the heterotetramers were conformationally less dynamic. To investigate subtype-specific antagonism, we engineered GluK2 and GluK5 mutants with altered affinities for the competitive antagonist UBP310. Structural analysis of these mutants revealed distinct UBP310 binding modes on the GluK2 versus the GluK5 subunits. Furthermore, we demonstrated that targeting GluK5 was more effective than targeting GluK2. Specifically, blocking GluK5 locked the receptor in a pore-occluded conformation, whereas antagonizing GluK2 retained structural flexibility above the pore. These findings provide a structural framework for understanding the distinct contributions of the GluK2 and GluK5 subunits to KAR function.

**Teaser:**

Cryo-EM structures of heteromeric kainate receptors reveal unique assembly and regulation mechanisms.

## Full Text Availability

The license terms selected by the author(s) for this preprint version do not permit archiving in PMC. The full text is available from the preprint server.

